# Diagnosis and treatment of heart failure in hereditary transthyretin amyloidosis

**DOI:** 10.1007/s10286-019-00629-5

**Published:** 2019-08-26

**Authors:** Gisela D. Puig-Carrion, Alex Reyentovich, Stuart D. Katz

**Affiliations:** Leon H. Charney Division of Cardiology, NYU Langone Medical Center, New York University Langone Health, 530 First Avenue, Skirball Suite 9 N, New York, NY 10016 USA

**Keywords:** Amyloidosis, Transthyretin, Heart failure, Diagnosis, Medical treatment

## Abstract

Amyloidosis describes a family of related disease states associated with the extracellular tissue deposition of fibrils composed of low-molecular-weight subunits of a variety of proteins circulating as constituents of plasma. Depending on the disease subtype, fibrillar deposits in a several organs including the heart, kidney, liver, and peripheral nerves cause organ dysfunction and associated morbidity and mortality. The most common amyloid fibril deposits associated with cardiac manifestations are of monoclonal light-chain or transthyretin (ATTR) types. This review will focus on the ATTR types of cardiac amyloidosis. ATTR amyloidosis may be associated with abnormal metabolism of wild-type transthyretin (previously called senile systemic amyloidosis) or with hereditary variants in the transthyretin gene. Cardiac amyloidosis is often under-recognized in its early stages, and when a diagnosis of cardiac amyloidosis is made, patients are often at the advanced stages of the disease. Treatments now available appear to exert their benefit predominantly in individuals with the early stages of disease. Increased awareness and early diagnosis of cardiac amyloidosis and continued discovery of effective therapies will increase opportunities to improve clinical outcomes in this patient population.

## Introduction

Amyloidosis is a group of related disorders characterized by the deposition of an extracellular proteinaceous material known as amyloid in the tissues [1, 2]. Tissue deposits occur as a result of misfolding of a precursor protein, the most common of which are light chains, transthyretin, and serum amyloid. Depending on the disease subtype, fibrillar deposits in the heart, kidney, liver, and/or the peripheral nerves cause organ dysfunction and associated morbidity and mortality. The heart may be the primary organ involved in amyloidosis of varying fibril types, or cardiac involvement may be present with lesser severity as a comorbidity of other primary organ involvement [3, 4]. In the past, the most commonly recognized form of cardiac amyloidosis was associated with plasma cell dyscrasias, with amyloid deposits derived from clonal light chains (AL amyloidosis). Once congestive heart failure symptoms become manifest in AL amyloidosis, the median survival is less than 6 months in untreated patients [5, 6]. Cardiac amyloidosis is also associated with mutant and wild-type forms of transthyretin (Amyloid Transthyretin Amyloidosis or ATTR) [3, 4]. The high population prevalence of the mutations in the transthyretin gene makes hereditary ATTR the most common form of familial amyloid cardiomyopathy and possibly the most common subtype of amyloid heart disease. Wild-type ATTR fibril deposition (formerly called senile amyloidosis) can also lead to heart failure (HF) and has been found to be potentially causative in one-third of patients with heart failure with a preserved ejection fraction [7]. In the hereditary subtype of ATTR cardiac amyloidosis, the specific site of an amino acid substitution determines the phenotype of the disease, which is transmitted as autosomal dominant with high penetrance, with clinical manifestations occurring most commonly after the age of 40 [2]. The cardiac manifestations in the hereditary forms of ATTR amyloidosis will be the primary focus of our review.

### Pathophysiology

Transthyretin is a circulating homotetramer carrier protein that binds to thyroid hormones and retinoic acid. The precursor protein ATTR in mutant form is responsible for the clinical manifestations of familial amyloidosis, whereas abnormal processing of wild-type ATTR can cause senile amyloidosis [8]. The presence of mutations leading to amino acid substitutions in ATTR destabilizes the tetramer, promoting amyloid fibril formation. Various ATTR mutations have different predilections for deposition in the heart and/or peripheral nervous system [7]. Different subtypes of cardiac amyloid can be characterized by the biochemical constituents of the fibril deposition, but tissue deposits share a similar histological appearance, and all share certain common biophysical components related to fibril formation. The source of the precursor proteins that form insoluble amyloid fibril deposits defines the nature of the disease in the individual patient [1, 2, 8, 9].

### Clinical presentation

Clinical manifestations of cardiac amyloidosis are largely determined by the class of precursor protein, tissue distribution of amyloid fibril deposition, and amount of amyloid fibril deposition [4, 8]. A thorough history and review of systems is essential for diagnosis, as noncardiac manifestations of amyloidosis may precede the onset of cardiac signs and symptoms. Noncardiac manifestations often associated with cardiac amyloidosis include waxy skin and easy bruising, macroglossia, gastrointestinal manifestations including hepatomegaly and diarrhea, impaired coagulation, renal dysfunction (decreased glomerular filtration rate and proteinuria), peripheral neuropathy (profound sensorimotor disturbances, with deterioration in activities of daily living such as ambulation), and autonomic nerve dysfunction (e.g., causing orthostatic hypotension, impotence, and urinary incontinence) [6, 10]. The detection of peripheral neuropathy without long-standing diabetes or other explanation should prompt consideration of evaluation for systemic amyloidosis. Amyloid fibril deposition is also associated with carpal tunnel syndrome and spinal stenosis [11]. AL or ATTR amyloid fibril deposition in tendinous tissues may manifest as bilateral carpal tunnel syndrome years before the onset of cardiac symptoms. Of patients with idiopathic carpal tunnel syndrome, 34% will have amyloid deposition in tenosynovial tissue; bilateral carpal tunnel disease should prompt consideration of further evaluation for detection of early ATTR cardiomyopathy [12]. Tenosynovial biopsy is a low-risk procedure that may lead to early diagnosis of amyloidosis, thereby allowing for timely intervention for treatment [13].

Amyloid infiltration in the heart may initially present with atrial fibrillation and conduction system disease, but due to the high prevalence of these conditions in the general population, the diagnosis of amyloidosis is frequently not considered [14]. As amyloid infiltration progresses, symptomatic heart failure is more commonly manifest, characterized by dyspnea with exertion, fatigue, peripheral edema, hepatomegaly, and ascites [3, 4]. Although left heart pressures in amyloid are elevated, frank pulmonary edema is uncommon. Suspicion of cardiac amyloidosis is increased with findings of left ventricular hypertrophy by echocardiography associated with low voltage on electrocardiography, and presence of conduction system abnormalities on electrocardiography. The degree of functional impairment may not be closely linked to the structural heart abnormalities seen on echocardiography, and varies according to amyloid subtype. Rapidly progressive congestive heart failure in AL amyloid is partly attributable to a direct myocellular toxicity of circulating free light chains, whereas transthyretin amyloid appears to mediate cardiac dysfunction predominantly due to the disruptive effects of amyloid infiltration on myocellular mechanotransduction [15]. Amyloidogenic light-chain toxicity has subsequently been confirmed in animal experiments [16].

Heart failure due to any cause is frequently associated with acquired autonomic dysfunction due to reduction in stroke volume and consequent unloading of arterial baroreceptors [17]. Reduced stroke volume is associated with reflex activation of the sympathetic nervous system, renin–angiotensin–aldosterone system, and arginine vasopressin system. Activation of these hormonal systems serves to increase systemic vascular resistance and preserve blood pressure in the setting of reduced cardiac output, but is also known to promote disease progression and increase long-term mortality risk [18]. Pharmacological inhibition of beta-adrenergic receptors and renin–angiotensin–aldosterone signaling reduce mortality in patients with heart failure associated with reduced ejection fraction, but are often associated with dose-limiting hypotension. Neurologic involvement in hereditary forms of ATTR amyloid disease can result in progressive sensorimotor neuropathy and autonomic neuropathy [6, 9]. In patients with cardiac amyloidosis and reduced stroke volume, acquired autonomic failure blocks the compensatory sympathetic vasoconstriction and increases the risk of symptomatic orthostatic hypotension. In mutations that lead to both neurologic and cardiac manifestations (e.g., ALA60), patients may develop profound debilitating orthostatic hypotension.

A variant of ATTR is found in patients of African-American descent; approximately 4% of this population is heterozygous for valine-to-isoleucine substitution at amino acid 122 (V122I) [7]. This mutation results in cardiac amyloidosis with onset of heart failure signs and symptoms typically after 60 years of age, but is not commonly associated with significant peripheral neuropathy manifestations. Observational data demonstrate that carriers of this mutation have greater risk for clinical manifestations of heart failure but not increased age-adjusted mortality [19]. The high prevalence of this mutation in the African-American population should raise suspicion for possible cardiac amyloidosis, even in the presence of hypertension, as an alternative explanation for left ventricular hypertrophy.

Common electrocardiographic changes associated with all forms of cardiac amyloidosis include low voltage, pseudoinfarct pattern, arrhythmias (atrioventricular block, atrial fibrillation, or less commonly ventricular tachycardia) [3]. Low voltage in limb leads is one of the most common abnormalities encountered in AL cardiac amyloid, but is less common in other forms of cardiac amyloidosis. All of these electrocardiogram abnormalities are nonspecific, so their primary value is to increase suspicion of disease and direct further diagnostic testing.

### Diagnostic testing

Cardiac amyloidosis can usually be diagnosed by histological confirmation of amyloid fibrils from noncardiac tissue in the context of supportive noninvasive cardiac imaging findings as determined by echocardiography, magnetic resonance imaging, or nuclear scintigraphy (bone avid tracers) [6, 20]. Nuclear imaging with technetium pyrophosphate tracer has sufficient specificity to definitively diagnose ATTR cardiac amyloidosis without tissue biopsy in the absence of evidence of monoclonal gammopathy.

Echocardiography is a noninvasive test with no ionizing radiation exposure that is commonly used in the diagnostic approach to cardiac amyloidosis [3]. Patients with both hereditary and wild-type forms of cardiac ATTR commonly manifest thickening of the left ventricle and free wall. Echocardiography in patients with cardiac amyloidosis may also demonstrate thickening of the valvular structures, right ventricular hypertrophy, left atrial enlargement, and normal to borderline reduced ejection fraction. Amyloid fibril deposition in the heart is associated with hypertrophy of the left ventricular wall and alterations in systolic and diastolic function of the left ventricle consistent with restrictive physiology. Amyloid infiltration in the myocardium increase diastolic chamber stiffness, with consequent abnormal diastolic filling and reduced end-diastolic volume [21]. Abnormalities in the diastolic filling pattern can be detected by Doppler ultrasound measurement of mitral valve inflow velocity; these diastolic function measures are prognostic in cardiac amyloidosis. Echocardiography-derived measures of myocardial strain (based on tissue Doppler ultrasound and speckle tracking) can detect early amyloid myocardial involvement. Relative apical sparing at two-dimensional speckle-tracking echocardiography has been reported to be specific for cardiac amyloidosis. Compared with mutant forms of ATTR, wild-type ATTR is generally characterized by greater left ventricular wall thickness, greater depression of ejection fraction, and higher longitudinal strain.

Evidence of systolic dysfunction (reduced ejection fraction and systolic strain abnormalities) occurs with a lesser degree of hypertrophy in patients with AL cardiac amyloidosis when compared with ATTR amyloidosis. Troponin and B-type natriuretic peptide (BNP) are useful cardiac biomarkers for assessing the severity of cardiac amyloidosis involvement.

Cardiac magnetic resonance imaging (cMRI) has the ability to distinguish cardiac amyloidosis from other forms of hypertrophic heart disease, due to a distinct pattern of myocardial nulling in late gadolinium enhancement over the subendocardial circumference [10, 22]. In advanced stages of cardiac amyloid disease, diffuse transmural late gadolinium enhancement also can be seen. Other typical cMRI findings include thickened ventricular walls with a normal left ventricular cavity size, right ventricular hypertrophy and dilation, and biatrial enlargement. Although not a common presentation, disproportionate accumulation of amyloid can be seen in the base of the ventricular septum, with dynamic left ventricular outflow obstruction leading to a misdiagnosis of hypertrophic (obstructive) cardiomyopathy.

Nuclear imaging of the heart with the tracer technetium-99m-labeled 3,3-diphosphono-1,2-propanodicarboxylic acid (^99m^Tc-DPD) has been compared with cMRI for assessing cardiac involvement. Ten years ago, in an attempt to differentiate AL amyloidosis from ATTR, the diagnostic accuracy of ^99m^Tc-DPD was investigated [23]. Using echocardiography as the reference standard for cardiac involvement, 100% sensitivity and specificity for ATTR was demonstrated, and DPD was proposed as a useful diagnostic component for distinguishing between forms of cardiac amyloidosis. cMRI and nuclear imaging have comparable capabilities for identification of myocardial amyloid deposition, but the infiltration burden can be markedly underestimated by visual analysis of cMRi compared with ^99m^Tc-DPD [24]. Cardiac uptake (grade 1, 2, or 3) on a radionuclide bone scan with ^99m^Tc-DPD is > 99% sensitive but not completely specific for cardiac ATTR amyloid (68% specificity compared with endomyocardial biopsy histology). Low specificity results largely from low-grade uptake in patients with cardiac AL or cardiac apolipoprotein A–I amyloidosis. Given these test diagnostic performance characteristics, bone scintigraphy alone provides accurate diagnosis of cardiac ATTR amyloidosis without the need for histology in patients who do not have evidence of monoclonal gammopathy [20].

**Fig. 1:**
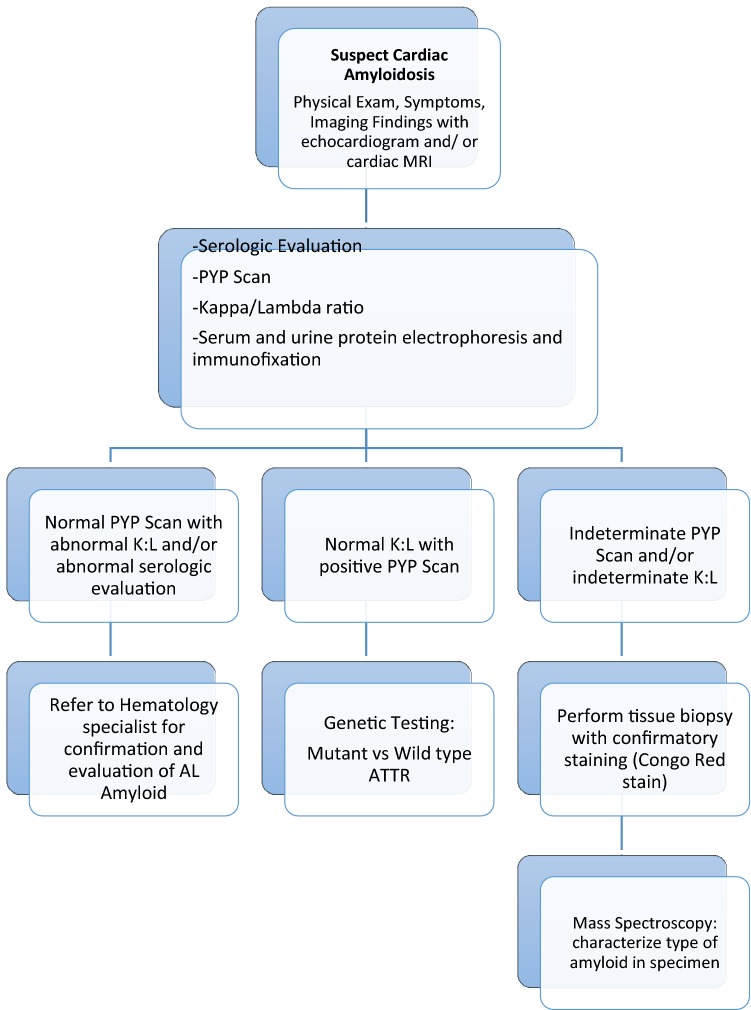
Algorithm for diagnostic approach

Tissue biopsy provides a gold-standard diagnosis of the presence and type of cardiac amyloidosis. Tissue biopsy that demonstrates apple-green birefringence when stained with Congo red under a polarizing microscope is required for the diagnosis of amyloidosis [3]. Sulfated Alcian blue, which is highly specific for amyloid, is used as an alternative stain. Fine-needle aspiration of the abdominal fat pad is a simple procedure that is positive for amyloid deposits in 70% of patients with AL amyloidosis. Nevertheless, unlike AL amyloidosis, in ATTR the abdominal fat aspirate frequently stains negative for amyloid, and endomyocardial biopsy may be necessary unless tissue is available for staining from a previous procedure such as carpal tunnel syndrome surgery [3, 10]. It is not necessary to perform endomyocardial biopsy if echocardiographic features are typical for cardiac amyloidosis and histological diagnosis has been made from other tissue [25, 26]. If the diagnosis is not confirmed by biopsy of other tissue, endomyocardial biopsy is extremely sensitive, as amyloid is widely deposited throughout the heart. A suggested algorithm for a diagnostic approach to suspected cardiac amyloidosis is summarized in Fig. 1.

### Treatment

Directed treatment options vary according to the type of amyloidosis. For example, therapy is directed at the underlying infectious or inflammatory disorder in secondary amyloidosis, whereas in AL amyloidosis, treatment is directed at the underlying plasma cell dyscrasia. Our main focus will be the treatment of transthyretin cardiomyopathy, which is a life-threatening disease, characterized by the accumulation of amyloid fibrils composed of misfolded transthyretin protein in the heart [27]. Median survival reported for untreated patients with symptomatic cardiac amyloidosis is 2.5 years after diagnosis of ATTR caused by the ATTR p.Val142Ile mutation and 3.6 years for wild-type ATTR [12, 28]. Death in most cases of ATTR cardiac amyloidosis is due to sudden cardiac death and progressive heart failure. Until recently, there have been no available evidence-based guideline-recommended treatments for ATTR amyloidosis, so therapy has been limited to supportive care and symptomatic relief based on expert consensus. Non-disease-modifying strategies include loop diuretics for those patients with cardiac amyloidosis who present with heart failure and volume overload. Diuretics must be titrated with caution, as excessive reduction of end-diastolic volume may induce symptomatic arterial hypotension. The addition of spironolactone to loop diuretic therapy may be considered to reduce symptomatic congestion and to reduce the likelihood of hypokalemia.

To maintain adequate blood pressure when the use of diuretics is needed, additional therapy with pharmacological agents that mediate vasoconstriction may reduce hypotensive symptoms. Midodrine forms an active metabolite which is an alpha-1 agonist activating receptors in the arteriolar and venous vasculature, resulting in an increase in vascular tone and blood pressure. l-threo-3,4-dihydroxyphenylserine (droxidopa), a prodrug that is metabolized to norepinephrine in nerve endings and other tissues, has been commercially available in Japan since 1989 for treating orthostatic hypotension symptoms in familial amyloid polyneuropathy [29]. Vasoconstrictive agents must be used with caution in patients with cardiac amyloidosis and limited contractile reserve, due to increased basal sympathetic activation in heart failure and potentially reduced cardiac output in response to increased afterload.

Beta-blockers, angiotensin-converting enzyme inhibitors, and angiotensin receptor blockers are considered standard therapy for most patients with heart failure and reduced ejection fraction, but should generally be avoided in patients with cardiac amyloidosis, regardless of left ventricular ejection fraction, due to high rates of intolerance and lack of evidence of clinical benefit.

Patients with cardiac amyloidosis can also present with both bradyarrhythmia due to heart block and tachyarrhythmia due to atrial and ventricular sources. Given the incidence of sudden death in patients with ATTR amyloidosis, prophylactic placement of an implantable cardioverter defibrillator can be considered to reduce the risk of sudden death in patients with cardiac amyloidosis and low ejection fraction, although without proven efficacy from controlled trials. Early insertion of a pacemaker can be helpful to relieve symptoms in patients with symptomatic bradyarrhythmias [10]. Atrial fibrillation is a common arrhythmia in patients with cardiac amyloid. Digoxin is a standard therapy for ventricular rate control in patients with heart failure, but should be used with caution in patients with cardiac amyloidosis. Digoxin binds to amyloid fibrils and can lead to high cardiac tissue levels and possibly increased risk of toxicity [30]. Amiodarone can be used to maintain sinus rhythm or slow atrioventricular conduction and is usually well tolerated. The role of catheter ablation for atrial arrhythmias in cardiac amyloidosis is uncertain.

According to the International Society for Heart and Lung Transplantation guidelines, heart transplants can be considered in patients without extra-cardiac manifestations of amyloidosis (Class IIa) [31]. Combined heart and liver transplant (eliminating pathologic transthyretin production) is also reasonable to consider in younger patients with hereditary forms of ATTR cardiac amyloidosis. In fact, the most common indication for combined heart and liver transplantation is amyloidosis (30% cases) [32]. Certain factors play a role in long-term survival after liver transplant, including age at onset, disease duration, mutation present, and nutritional status. It remains unclear whether early liver transplantation before the development of cardiomyopathy makes heart transplant unnecessary for specific mutations. Heart transplantation is generally not an option for older patients with wild-type ATTR cardiac amyloidosis.

Treatments for hereditary ATTR amyloidosis target protein suppression, with new studies indicating that lowering ATTR levels may be a successful approach to therapy. RNA interference (RNAi) is an endogenous mechanism for controlling gene expression, resulting in cleavage of target messenger RNA (mRNA) by small interfering RNAs bound to the RNA-induced silencing complex [33]. Patisiran, a hepatic-directed investigational RNAi therapeutic agent, reduces the production of mutant and wild-type ATTR by targeting the 3′ untranslated region of ATTR mRNA [34]. Patisiran has been found to improve neuropathy in patients with hereditary ATTR amyloidosis and has shown extended effects across the sensorimotor and autonomic domains, resulting in improved quality of life, walking, and nutritional status [33]. Polyneuropathy disability scores were also improved, including transition from assisted to unassisted walking, which is consistent with the effect of patisiran on gait speed. There was also evidence that patisiran improved cardiac manifestations of hereditary ATTR amyloidosis, as indicated by echocardiographic measures of cardiac structure and function, and a reduction in NT-proBNP levels [33, 35]. A post hoc safety analysis demonstrated that patisiran therapy was associated with a 46% reduction in cardiovascular hospitalization and death when compared with placebo [10.1 vs. 18.7 events per 100 patient-years, relative risk 0.54 (95% confidence interval 0.28–1.01)].

Antisense oligonucleotide drugs have been developed as an alternative therapeutic strategy for suppression of hepatic synthesis of ATTR. For ATTR, an antisense oligonucleotide that is exactly complementary to the messenger ribonucleic acid molecule that encodes ATTR is used; after the drug binds the messenger ribonucleic acid molecule, protein production may be inhibited by ribonuclease H-mediated destruction of the mRNA. Thus, the antisense drug can prevent or dramatically reduce ATTR production [10]. Inotersen (formerly IONIS-TTR/ISIS 420915) is a 2′-*O*-methoxyethyl-modified antisense oligonucleotide inhibitor of the hepatic production of transthyretin protein [36]. In a randomized double-blind placebo-controlled clinical trial, this inhibitor modified the course of neuropathy and improved quality of life in patients with hereditary transthyretin amyloidosis, demonstrating significant treatment benefits independent of ATTR mutation type, disease stage, or cardiomyopathy status at baseline. In a small open-label observational study of 15 patients with ATTR cardiac amyloidosis (8 familial and 7 wild-type), 12 months of therapy with inotersen was associated with improved cardiac structure and function, improved submaximal exercise capacity, and decreased levels of brain natriuretic peptide biomarker when compared with baseline [37].

Transthyretin is a 127-amino-acid protein primarily synthesized in the liver that transports thyroxine and retinol-binding protein–retinol (vitamin A) complex [38]. When the tetrameric structure of transthyretin protein dissociates into intermediates, fibrillogenesis occurs, misassembling into soluble oligomers, protofilaments, and amyloid fibrils. Kelly and colleagues discovered that a polymorphism in ATTR that encodes the amino acid substitution Thr119Met stabilized the protein in the context of a destabilizing pathogenic variant (Val30Met), leading to the development of tafamidis, a benzoxazole derivative lacking nonsteroidal anti-inflammatory drug activity that binds to the thyroxine-binding sites of transthyretin with high affinity and selectivity and inhibits the dissociation of tetramers into monomers [38]. This drug has been shown to slow the progression of peripheral neurologic impairment in ATTR amyloid polyneuropathy. In patients with wild-type and mutant ATTR cardiomyopathy, tafamidis treatment in an open-label trial stabilized ex vivo urea-mediated ATTR tetramer dissociation and echocardiographic indexes of left ventricular filling and systolic function [7, 39]. The Transthyretin Amyloidosis Cardiomyopathy Clinical Trial (ATTR-ACT), designed to determine the efficacy and safety of tafamidis in patients with hereditary and wild-type ATTR amyloid cardiomyopathy [40], showed that tafamidis reduced the combination of all-cause mortality and cardiovascular-related hospitalizations when compared with placebo [38]. Tafamidis also slowed the decline in functional capacity and quality of life over time when compared with placebo.

The nonsteroidal anti-inflammatory drug diflunisal stabilizes the ATTR tetramer, with evidence of clinical efficacy in studies in patients with familial amyloid polyneuropathy. A randomized clinical trial reported in 2013 showed reduced progression of neurological impairment and preserved quality of life 2 years after randomization to therapy with diflunisal; however, no information was provided regarding the cardiac response [41]. The clinical utility of diflunisal in ATTR cardiac amyloidosis may be limited by its potential to promote sodium retention and renal dysfunction in patients with more advanced heart failure.

In ATTR amyloid cardiomyopathy, the dissociation of the ATTR tetramer at the T-binding interface generates dimers that rapidly dissociate into amyloidogenic monomers, the rate-limiting step during ATTR misfolding and amyloid formation [42]. In a recent report by Penchala and colleagues, a potent and selective stabilizer of ATTR (AG10) prevented the dissociation of V122I ATTR in serum samples from patients with familial amyloid cardiomyopathy. AG10 also increased the stability of wild-type ATTR in human serum against acid-mediated dissociation that accelerates amyloidogenesis. The oral bioavailability of AG10, which has been used in rodents, makes it a suitable agent to treat ATTR amyloid cardiomyopathy. A recently published phase 2 trial demonstrated that AG10 treatment was well tolerated in patients with either mutant or wild-type ATTR cardiomyopathy, achieved target plasma concentrations, and demonstrated near-complete stabilization of ATTR. AG10 has the potential to be a safe and effective treatment for patients with ATTR cardiomyopathy; a phase 3 trial is currently under way [43].

In vitro studies suggest that epigallocatechin gallate, the most abundant catechin in green tea, inhibits amyloid fibril formation. One study described 19 patients with ATTR cardiomyopathy who were serially evaluated with echocardiography and CMRi after consuming green tea or green tea extracts [44]. After 12 months, no increase in left ventricular wall thickness or left ventricular mass was observed by echocardiography, suggesting that green tea or its extracts might inhibit the progression of cardiac amyloidosis.

Doxycycline can also be used as therapy, since several studies have proven that it can disrupt fibrils, and its treatment in a mouse model of amyloidosis resulted in amyloid disaggregation and improvement in some tissue markers associated with ATTR deposition [45]. A more pronounced, synergistic effect on the removal of tissue ATTR deposits was observed when doxycycline was administered in combination with the antiapoptotic agent tauroursodeoxycholic acid. On the basis of preclinical studies, a phase II open-label study was designed to evaluate the efficacy, tolerability, safety, and pharmacokinetics of doxycycline and tauroursodeoxycholic acid, with preliminary data supporting a beneficial effect and an acceptable toxicity profile [46].

## Conclusion

Advances in diagnosis and treatment have transformed cardiac amyloidosis from an uncommon and untreatable disease with a universally fatal outcome, to a potentially treatable and more common form of hypertrophic heart disease. Early recognition is key for optimizing success of disease-modifying therapeutics. If typical imaging findings are present without evidence of monoclonal gammopathy, a nuclear study of the heart (pyrophosphate scan) can confirm diagnosis of ATTR. Disease-modifying treatment includes liver transplant and investigational therapies designed to suppress hepatic synthesis, stabilize ATTR proteins, and prevent misfolding. In selected patients with ATTR cardiac amyloidosis, heart transplant or combination of heart/liver transplant can be curative. Pharmacological therapies aimed at stabilizing or suppressing the formation of ATTR are a promising avenue for future investigations.
